# Impact of Channels Aspect Ratio on the Heat Transfer in Finned Heat Sinks with Tip Clearance

**DOI:** 10.3390/mi13040599

**Published:** 2022-04-12

**Authors:** Elena Martin, Alejandro Valeije, Francisco Sastre, Angel Velazquez

**Affiliations:** 1 Departamento de Ingeniería Mecánica, Máquinas y Motores Térmicos y Fluidos, Escuela de Ingeniería Industrial, Campus Marcosende, Universidade de Vigo, 36310 Vigo, Spain; alexvaleije@uvigo.es; 2 Fluid Mechanics and Aerospace Propulsion Department, Universidad Politécnica de Madrid, Plaza del Cardenal Cisneros 3, 28040 Madrid, Spain; francisco.sastre@upm.es (F.S.); angel.velazquez@upm.es (A.V.)

**Keywords:** heat sink, tip clearance, aspect ratio, heat transfer versus pressure drop, numerical simulation, Computational Fluid Dynamics (CFD)

## Abstract

A 3D numerical study is used to analyze the flow topology and performance, in terms of heat transfer efficiency and required pumping power, of heat sink devices with different channel aspect-ratio in the presence of tip-clearance. Seven different channel aspect ratios AR, from 0.25 to 1.75, were analyzed. The flow Reynolds numbers Re, based on the average velocity evaluated in the device channels region, were in the range of 200 to 1000. Two different behaviors of the global Nusselt were obtained depending on the flow Reynolds number: for Re<600, the heat transfer increased with the channels aspect ratio, e.g., for Re=400, the global Nusselt number increased by 14% for configuration AR=1.75 when compared to configuration AR=0.25. For Re>600, the maximum Nusselt is obtained for the squared-channel configuration, and, for some configurations, flow destabilization to a unsteady regime appeared. For Re=700, Nusselt number reduced when compared with the squared-channel device, 11% and 2% for configurations with AR=0.25 and 1.75, respectively. Dimensionless pressure drop decreased with the aspect ratio for all cases. In the context of micro-devices, where the Reynolds number is small, these results indicate that the use of channels with high aspect-ratios is more beneficial, both in terms of thermal and dynamic efficiency.

## 1. Introduction

One big challenge in thermal engineering applications is the potential advantage of implementing tip clearances in heat sink configurations to reduce pressure drop in the system, without carrying a large penalty in the heat transfer. This would be beneficial for multiple heat sink configurations using single-phase, two-phase, phase-change and/or porous materials [1,2,3]. In small environments where pumping power is a limiting factor and cannot be increased at will, like in avionics systems, reducing pressure drop is critical, even if it comes with a cost of reducing the heat transfer performance.

The first article published in this field was this from Sparrow, Baliga and Patankar [4] in 1978. In this article, a shrouded fin array is studied with and without tip clearance. Later, in [5], Sparrow and Kaddle used the same geometry from an experimental perspective. There are plenty of articles in journals which deal with this subject. In 2011, Reyes et al. [6] carried out an experimental study on the effects of tip clearance on a micro-channel based heat sink with squared channels. In this study, three different values for the clearance height were considered and compared with the referenced case with no clearance present. The Reynolds number varied between 400 and 2600 based on the hydraulic diameter of the flow passages. For this work, working fluid was water and heat sink wall temperature was kept at a constant 70 °C. Authors found that, for the lowest Re, both pressure drop and heat transfer were very sensitive to the tip clearance. Optimum configuration was found when the tip clearance was equal to the fin height (500 μm). For this case, the Nusselt number (Nu) achieved 83% of the value for the reference case, while the pressure drop (ΔP) decreased to a 20% of the reference case. However, for the highest Re, it was observed that both heat transfer and pressure drop showed a weak dependence on the tip clearance. More specifically, heat transfer degradation and pressure drop improvement rates at the highest Re were similar regardless of the tip clearance height. In 2014, Mei et al. [7] studied the effect of tip clearance in micro-pin type geometries and reported heat transfer degradation rates of 50% with pressure losses reductions by a factor of about 4. In 2016, Liu et al. [8] made an experimental study on the influence of the tip clearance in a group of micro-cylinders placed on a heat sink. Authors focused on a low Reynolds regime and, for Re=400 and the optimum clearance, they reported a 15% reduction of heat transfer and a 50% pressure drop compared to their reference case. Li [9] published an experimental study that accounts for, among other things, the influence that tip clearances have on the thermal performance of pin-fin arrays. In this study, authors were able to find linear correlations between thermal performance, the Reynolds number and tip to outer surface distance. Jadhav and Balaji [10] presented another experimental study where they reported in their conclusions that the effect of tip clearance on thermal performance is more pronounced at lower flow velocities. Giri and Das [11] made a computational study where they considered a shrouded rectangular fin array, varying clearance spacing, fin spacing and Reynolds number. Sastre et al. [12] studied a squared-channel fin configuration with tip clearance both experimentally and numerically. They reached the conclusion that heat transfer was greatly reduced by the presence of the tip clearance, suggesting that small tip clearances, which are able to significantly reduce the pressure drop, are more convenient to keep a good heat transfer performance.

Concerning the effect of the channels aspect ratio on the heat transfer performance, the recent review of Zhou et al. [13], deals, among others, with the influence on the heat transfer performance and pressure drop of the aspect ratio of single-phase and two-phase fluids in micro-channel pipes. Several works cited in [13] indicate that the square-channel has a much better performance than other shape of channels (70% higher than circular-shape) [14,15] although other works (Vinoth and Kumar [16]) point to trapezoidal channel cross-sections as optimal. Brinda et al. [17] found that heat transfer coefficient could be enhanced by smaller aspect ratio, while Wang et al. [18] indicate that high aspect ratio rectangular channels perform better. The best performance was obtained for aspect ratios between 8.9–11.5.

Pan et al. [1] investigated numerically different aspect ratios with different fluid and materials for fixed mass flow rate. The results show that the heat transfer performance of the manifold microchannel heat sink reaches the peaks at one specific channel aspect ratio whose value depend on the working fluid and solid material. Wu and Zhang [19] evaluated the heat transfer capacity of Al${}_{2}$O${}_{3}$-Water nanofluids in microchannels of different aspect ratios. They conclude that increasing the aspect ratio of the microchannel effectively improved the heat transfer capacity of the heat sink without significantly increasing the flow resistance loss. They also observed a limiting value of the heat transfer for aspect ratio equal to 30. Parlak et al. [20] selected an aspect ratio of 0.01 for providing both the lowest power consumption and sufficient cooling. They observed that an increase in the aspect ratio up to a certain value results in a lower pressure drop and higher thermal performance. Ma et al. [21] researched numerically the sensitivity of flow and heat transfer process to different design parameters in typical rectangular microchannels. They conclude that the number of channels and the Reynolds number have a greater impact on heat transfer performance under low Re conditions, while the cross-sectional area and aspect ratio have a much higher effect on the pumping power rather than Nusselt numbers. They also observed that for high Reynolds, the number of channels became the dominant factor that influenced both heat transfer and flow performance of the microchannel heat sink.

To sum up, it can be said that there is a qualitative tendency in all the above mentioned references regardless of the type of geometry under consideration. Namely, the main effect of the tip clearance is to reduce the pressure drop significantly while, at the same time, degrading the heat transfer. On the other hand, the aspect ratio of the channels has a significant impact on the microchannel heat transfer performance. However, the actual quantification of these effects seems to be very dependent on the studied problem, somewhat contradictory and not studied yet in detail in the presence of tip clearance. In this context, the objective of the present study consists on conducting three dimensional numerical simulations of a simplified general heat sink configuration with tip clearance (similar to [12]) with straight fins of different aspect ratios, to determine the flow topology and thermal performance for different flow conditions. The main novelty of the work that is being presented is that the effect of tip clearance geometry is quantified by means of performing a systematic parameter analysis. The parameters that have been considered are: ratio of tip clearance to channel width, ratio of channel height to channel width and Reynolds number, respectively.

## 2. Mathematical Model

**Figure 1:**
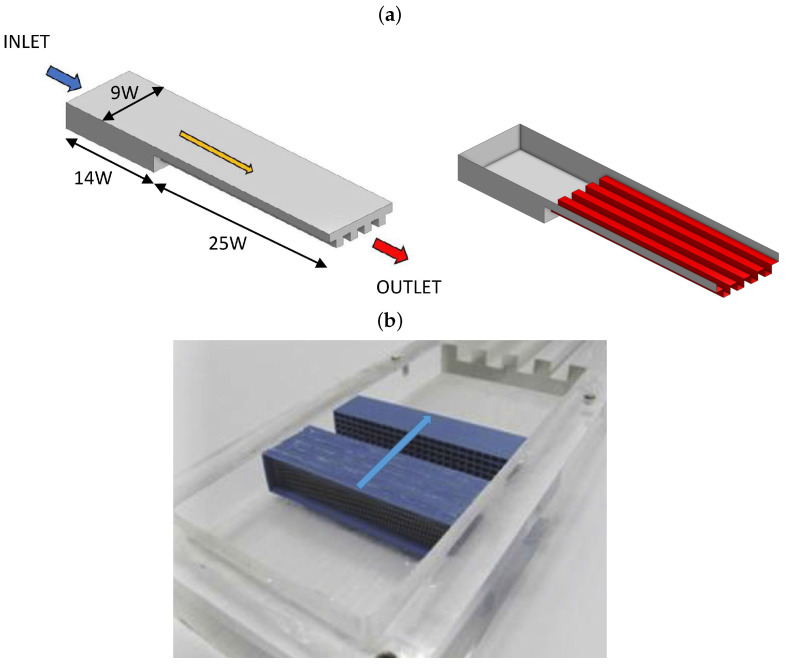
(**a**) Sketch of the domains dimensions and detail of the heated (red color) fin walls. (**b**) View of the experimental prototype (lid was retired for the picture), of dimension *W* = 10 mm, used to validate the numerical model.

**Figure 2:**
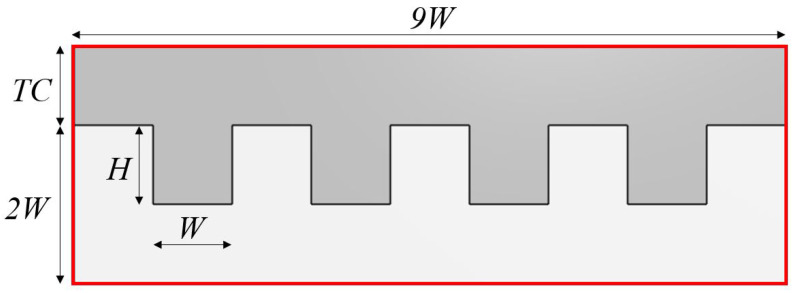
Sketch of the inlet cross section (outlined with red colored lines) and the flow cross section in the channels region (gray color).

The problem analyzes the confined flow of liquid water at low to moderate Reynolds number inside different multi-channel devices, as shown in Figure 1. The device is formed by four heated rectangular channels (or fins), of fixed width *W*, downstream connected to a chamber. The device has a tip clearance (TC), measured between the top part of the fins and the lid of the device (see Figure 2), aimed to significantly diminish the pressure drop in the heat sink. The connection between the chamber and the channels is made by a forward step-like structure of height equal to 2W. Thus, the dimensions of the device inlet section, outlined in red in Figure 2, is $A_{inlet}=9W\times \left(T\;C+2W\right)$. The flow cross-section in the channels region, shaded in gray color in Figure 2, is $A_{channels}=9W\;T\;C+4HW$, where *H* is the channels height. Four different tip clearances ratios,

(1) TR=TC/W=0.5,1,1.5,and2,

were considered. Additionally, seven different aspect ratios (AR) of the channels, defined by the ratio of the channel height *H* and width *W*:

(2) AR=H/W=0,0.25,0.5,0.75,1.0,1,25,1.5,and1.75,

were analyzed in this study. To limit the number of simulated configurations, channels aspect ratio AR was varied for a fixed tip clearance ratio of TR=0.5.

The problem can be described by the following unsteady incompressible Navier-Stokes and heat transfer Equations (3)–(5):

(3) $$\tilde{\nabla }\cdot \tilde{v}=0$$

(4) $$\frac{\partial \tilde{v}}{\partial \tilde{t}}+\tilde{v}\cdot \tilde{\nabla }\tilde{v}=-\tilde{\nabla }\tilde{p}+\frac{1}{Re}\tilde{\nabla }\cdot \left(\tilde{\mu }\left(\tilde{T}\right)\left[\tilde{\nabla }\tilde{v}+\tilde{\nabla }{\tilde{v}}^{T}\right]\right)$$

(5) $$\frac{\partial \tilde{T}}{\partial \tilde{t}}+\tilde{v}\cdot \tilde{\nabla }\tilde{T}=\frac{1}{Re\;Pr}\tilde{\nabla }\cdot \left(\tilde{k}\left(\tilde{T}\right)\tilde{\nabla }\tilde{T}\right)$$

The fluid velocity, the spatial coordinates *x*, *y* and *z* (streamwise, spanwise and vertical coordinates, respectively), the computational domains, time, temperature differences and hydraulic pressure, have been rendered dimensionless using, respectively: the mean fluid velocity $U_{\infty }$ in the channels section, the hydraulic diameter $D_{h}$ of the section in the channels regions, the time $D_{h}/U_{\infty }$, the difference of temperature between the heated wall and the inlet $T_{w}-T_{\infty }$ and the inlet fluid density $\rho_{\infty }$:    

$$\begin{array}{ccc} \tilde{v} & = & \left(\tilde{u},\tilde{v},\tilde{w}\right)=\left(\frac{u}{U_{\infty }},\frac{v}{U_{\infty }},\frac{w}{U_{\infty }}\right)\\ \tilde{x} & = & \left(\tilde{x},\tilde{y},\tilde{z}\right)=\left(\frac{x}{D_{h}},\frac{y}{D_{h}},\frac{z}{D_{h}}\right)\\ \tilde{t} & = & \frac{t}{D_{h}/U_{\infty }}\\ \tilde{T} & = & \frac{T-T_{\infty }}{T_{w}-T_{\infty }} \end{array}$$

(6) $$\tilde{p}=\frac{p}{\rho_{\infty }U_{\infty }^{2}}$$

The hydraulic diameter of the transverse section where the channels are located is obtained as follows:

(7) $$\begin{array}{c} D_{h}=\frac{4A_{channels}}{Perimeter}=2W\frac{9T\;R+4A\;R}{9+T\;R+4A\;R} \end{array}$$

As can be seen, $D_{h}$ depends on the tip clearance ratio and the aspect ratio, so it changes for each configuration. The rationale of selecting this length to make the problem dimensionless is to compare, for a given size of the channels section, which configuration is more efficient.

Reynolds and Prandtl numbers are then defined as $Re=\frac{\rho_{\infty }U_{\infty }D_{h}}{\mu_{\infty }}$ and $Pr=\frac{\mu_{\infty }\;c_{p_{\infty }}}{k_{\infty }}$, where $\mu_{\infty }$, $k_{\infty }$ and $c_{p_{\infty }}$ stand for the fluid viscosity, thermal conductivity and specific heat evaluated at the inlet. For the simulated cases, Pr was equal to 4.8. $\tilde{\mu }$ and $\tilde{k}$ stand for the temperature dependent dimensionless viscosity and thermal conductivity of the fluid. Since the problem has been defined to be studied with water as the working fluid, the empirical correlations for these values have been taken from the works of Martin and Velazquez [22] and Martin et al. [23] and made dimensionless accordingly.

Simulated Reynolds numbers fall in the range of [200–1000]. As the intended application for this device is to be used as a heat sink in micro-scales with water, buoyancy effects can be neglected in the model. Maximum temperature variations considered in the thermal problem were equal to 35 °C (for example, having water at 35 °C at the inlet and at 70 °C at the heated walls, as in the experimental work of Reyes et al. [6]). Therefore, density gradients in the continuity Equation (3) were neglected.

### 2.1. Boundary Conditions

For the normal inlet velocity and temperature, the following uniform values were set:

(8) $${\tilde{u}}_{inlet}=U_{\infty }\frac{A_{channels}}{A_{inlet}}=U_{\infty }\frac{9T\;R+4A\;R}{9T\;R+18},\;{\tilde{T}}_{inlet}=0$$

while zero normal gradient was imposed for the pressure. The inlet velocity conditions were validated by conducting isothermal experiments in devices with *W* = 10 mm for the different TR and comparing the measured velocities (using Particle Images velocimetry PIV techniques) with the computational results. Details of the experimental setup and validation are described in [12]. For all the device walls, a non-slip condition for the velocity and zero normal pressure gradient was used. For the heated walls (red surface of Figure 1), a uniform temperature boundary condition ${\tilde{T}}_{w}=1$ was imposed. The rest of the device walls are considered adiabatic. Zero normal gradients for the velocity components and temperature, as well as reference pressure equal to $\tilde{p}=0$ were used at the outlet. The problem was initialized using the solution obtained using a potential solver starting from initial uniform zero pressure, zero velocity and zero uniform temperature.

### 2.2. Quantification of Heat Transfer

To quantify the local and global heat transfer between the heated walls (of surface $A_{w}=25W^{2}\left(9+8A\;R\right)$) and the fluid, the following local and global Nusselt numbers were defined and calculated numerically at the heated walls:

(9) $$N\;u=\frac{q_{w}}{k_{\infty }\frac{T_{\infty }-T_{w}}{D_{h}}}=\tilde{k}\left(\tilde{T}\right)\frac{\partial \tilde{T}}{\partial \tilde{\eta }}$$

(10) $$\bar{N\;u}=\frac{Q_{w}}{k_{\infty }\frac{T_{\infty }-T_{w}}{D_{h}}A_{w}}=\frac{D_{h}^{2}}{A_{w}}\int_{{\tilde{A}}_{w}}\tilde{k}\left(\tilde{T}\right)\frac{\partial \tilde{T}}{\partial \tilde{\eta }}d{\tilde{A}}_{w}$$

where $q_{w}$ and $Q_{w}$ are the dimensional net heat transfer flux and heat transfer rate between the heated walls and the fluid, respectively. For convenience, dimensionless pressure losses across the device $\Delta \tilde{p}={\tilde{p}}_{inlet}-{\tilde{p}}_{outlet}$ will be also computed for each case as it is a parameter that conditions the required pumping power: $Power=\Delta \tilde{p}\;\rho_{\infty }U_{\infty }^{3}A_{channels}$.

### 2.3. Numerical Implementation

The numerical solver was based on a finite volume formulation [24] solved with the open source CFD software OpenFOAM (www.openfoam.org, accessed on 11 April 2022). Standard solver pisoFoam, which is used to simulate laminar, unsteady flows without heat transfer, was adapted to calculate the heat flow equation and additional functions, such as the thermal variation of the fluid viscosity and conductivity in each iteration. As its name suggests this solver uses the pressure-velocity PISO (Pressure-Implicit with Splitting of Operators) coupling algorithm to generate a pressure field that fulfils the continuity Equation (3). The under-relaxation factor used in this work was 0.3 [25], while the other solution variables were under-relaxed by a factor 0.7 to accelerate convergence. The selected spatial discretization schemes for each term of the model were all second order. The segregated linear systems obtained upon discretization of the problem were solved with Geometric Algebraic Multi Grid iterative method for pressure and temperature and smoothed Gauss-Seidel for velocity. Visualization of the computed fields and computation of global Nusselt number and pressure drop was carried out using open-source, multi-platform data analysis and visualization application ParaView (www.paraview.org, accessed on 11 April 2022).

To validate the implementations introduced in the standard solver to include the heat transfer equation and the properties variation with temperature, numerical tests were performed to contrast the results with the work of Lyczkowski et al. [26]. The geometry tested consisted in a square channel of 10 mm height and 500 mm long with a fully developed flow inlet boundary condition under an inlet Reynolds number of 100 based on the channel hydraulic diameter and an inlet temperature of 35 °C. The channel walls were at a constant temperature of 70 °C. The working fluid used was water and the properties were constant. For this case, the global Nusselt number was 2.98, in concordance with the results shown in [27], and the Nusselt number at a dimensionless distance from the inlet ($x/D_{H}Re_{D_{H}}Pr$) of 0.07 was 2.79, which is in very good agreement with the results in the work of Lyczkowski.

**Figure 3:**
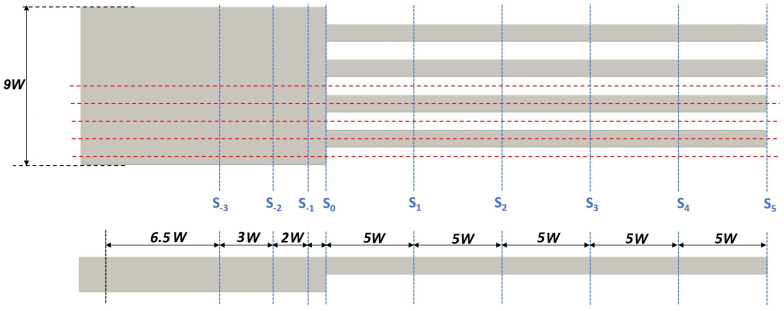
Location of transversal study planes *S*.

Uniform structured meshes with cell sizes of $\Delta \tilde{x}=0.05$ and $\Delta \tilde{y}=\Delta \tilde{z}=0.02$ for each domain were generated, leading to meshes with 23.5 million of cells for configurations with TR=0.5 and AR=0.25 and 72 million for TR=0.5 and AR=1.75. An adaptive time step was selected by the solver to ensure a maximum Courant number smaller than 0.75 for all the mesh cells. In order to select the appropriate mesh size (and associated time-step), a sensitivity analysis was performed with two coarser meshes for configuration TR=AR=1 and Re=828. The variables to compare were the deviation $\varepsilon_{1}$ of the streamwise velocity profile at section $S_{-1}$, located nearly the end of the chamber section (see Figure 3), and the deviation of the dimensionless total heat transfer $\varepsilon_{2}$ obtained at the heated walls. Sensitivity results in Table 1 show that, although Mesh #2 leads to acceptable results, Mesh #3 was selected because it provided better accuracy with acceptable simulation times.

## 3. Results and Discussion

**Figure 4:**
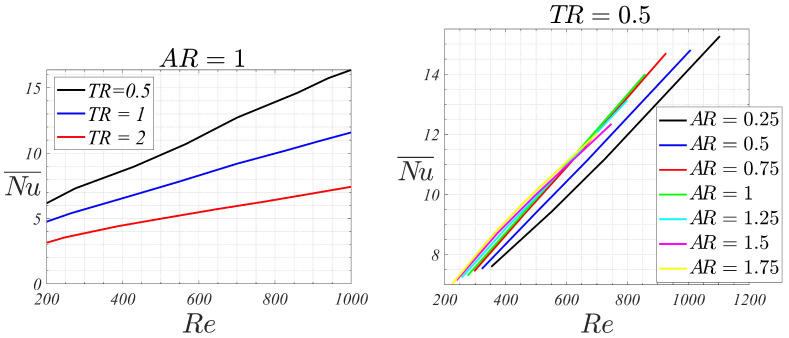
$\bar{Nu}$ vs. Re. (**Left**) for different tip clearance ratios TR (with AR=1). (**Right**) for different aspect ratios AR (with TR=0.5).

Global Nusselt number dependency on Re, TR and AR is shown in Figure 4. It can be observed that dependency of the global Nusselt number with Reynolds number follows approximately a linear trend for the studied region, which is in accordance with the correlations shown in [28] for rectangular microchannels in laminar flow. Given the same Reynolds, left Figure 4 shows that increments of the tip clearance ratio TR diminish both the overall Nusselt number and the slope of the $\bar{Nu}-Re$ curve. For example, for Re=700, $\bar{Nu}$ is equal to 13, 9.2 and 6.1 for TR 0.5, 1 and 2, respectively. That means, that doubling the tip clearance from 0.5 to 1, the Nusselt number is reduced a 28%, while doubling tip clearance from 1 to 2, the reduction in the Nusselt number is 38%. Therefore, the presence of the tip clearance, which reduces significantly the pressure drop and, consequently, the needed pumping power, degrades greatly the heat transfer efficiency.

A limiting case that could be considered for comparison purposes is the one corresponding to AR = 0 (*H* = 0). This case involves a rectangular section channel with no fins present. Thereby, since it addresses the simplest channel geometry, it could be used to quantify the practical effect of actually implementing fins on the most basic geometry. When assuming laminar flow, the Nusselt number in a rectangular channel can be obtained by analytical means, see for example [29]. In practice, the ensuing Nusselt numbers (that do not depend on the Reynolds number in the laminar regime) are tabulated as a function of the rectangular channel aspect ratio. In this limiting case, in the problem under consideration, channel height and width are TC and 9W respectively (see Figure 2). Then, the channel aspect ratio is TC/(9W)=TR/9. For some representative values of TR considered in this study (0.5, 1 and 2) the associated Nusselt numbers are: 7.5, 5.6 and 4.4, respectively. As it could be observed in the right plot of Figure 4, Nusselt numbers associated to the finned geometry are higher than those values for Reynolds numbers larger than about 300.

Nevertheless, the aspect ratio AR of the channels has a more complex effect on the heat transfer than the tip clearance ratio. For small values of the channels aspect ratio, that is, for AR 0.25 up to 0.75, the Nusselt number increases linearly with the flow Reynolds number. Additionally, the Nusselt is higher for higher channel aspect ratio, and the slope of the $\bar{Nu}$-Re line also increases, though marginally, with the aspect ratio.

**Figure 5:**
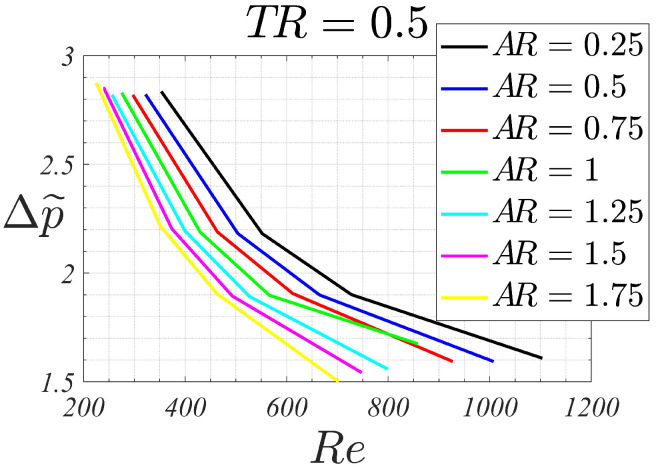
Pressure drop $\Delta \tilde{p}$ vs. Re for different aspect ratios AR, with TR=0.5.

For AR=1, the $\bar{Nu}$-Re line collapses practically with the corresponding for AR=0.75, still following a linear behavior. For the highest aspect ratios (AR between 1.25 to 1.75) deviations from the linear trend are observed. Nusselt numbers are slightly higher than those for AR=1 up to Re∼600, while for higher Reynolds numbers, smaller Nusselt numbers (than the corresponding for squared-channels) are obtained. This indicates that, for high aspect ratio channels configurations and high Reynolds, heat transfer efficiency degrades, with the squared channel being the most efficient configuration. However, for moderate Reynolds, smaller than 600, the heat transfer efficiency is slightly higher for high aspect ratio channels, meaning that these are the optimal configurations in this low Reynolds regime. Moreover, these high aspect ratio channel configurations are more beneficial in terms of pressure drop. Figure 5 shows the dimensionless pressure drop $\Delta \tilde{p}$ for the different channels aspect ratios. For a given Reynolds number, the higher the channel aspect ratio AR, the smaller the dimensionless pressure drop is. As pumping power scales proportionally to $\Delta \tilde{p}\;\rho_{\infty }U_{\infty }^{3}A_{channels}$, at equal velocity and cross-sectional area, a device with higher AR would require smaller pumping powers.

As an example, for Re=400, the Nusselt changes from 8.5 to 9.7, for AR 0.25 to 1.75, respectively, which means that the heat transfer for the highest aspect ratio device has increased to 14% when compared to its counterpart with the lowest aspect ratio. For Re=700, the corresponding Nusselt for AR 0.25, 1 and 1.75 are 10.9, 12.2 and 12, respectively. Therefore, the maximum heat transfer is obtained for the squared-channel device, and the heat transfer reduces 11% for the smallest aspect ratio configuration and 2% for the highest.

**Figure 6:**
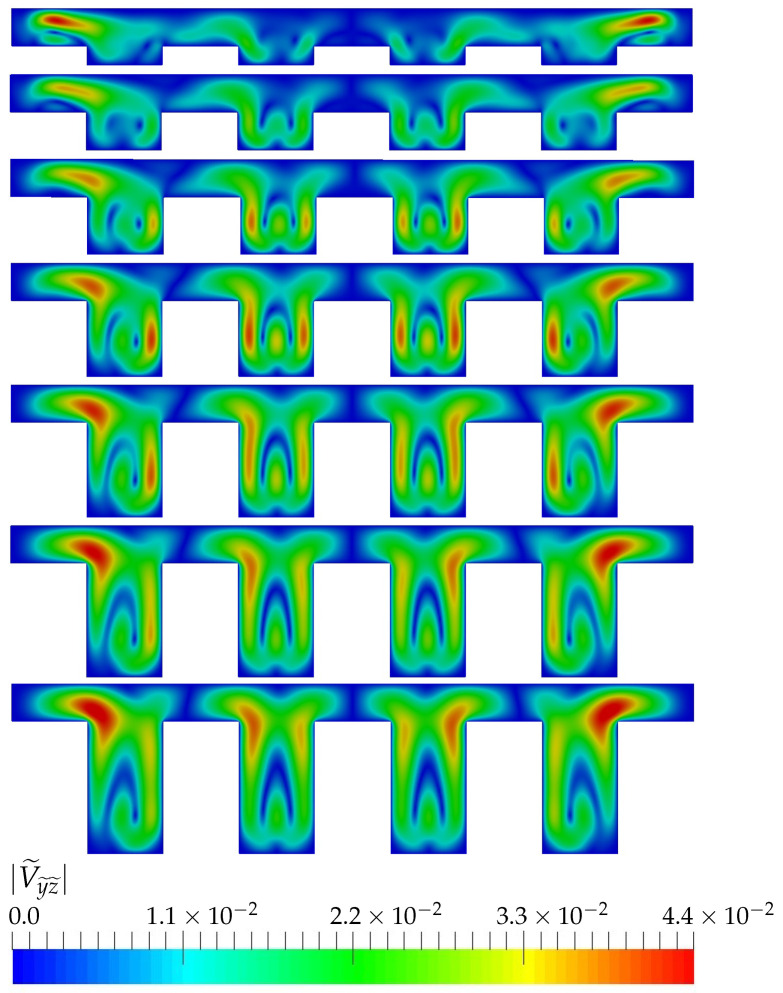
Transverse velocity at plane $S_{1}$ for Re=400, TR=0.5 and different AR.

Local topology of the flow for Re=400 is visualized in Figure 6, plotting the absolute value of the (transverse) velocities components in the directions ($\tilde{y}$ and $\tilde{z}$) at the transverse plane $S_{1}$ of the device (see plane location in Figure 3):

(11) $$|{\tilde{V}}_{\tilde{y}\tilde{z}}|=\frac{\sqrt{{\tilde{v}}^{2}+{\tilde{w}}^{2}}}{{\tilde{U}}_{\infty }}$$

As each device has different ${\tilde{U}}_{\infty }$, the transverse velocity in Equation (11) has been divided by the stream-wise average velocity of the cross-section in order to compare the local topology of the flow.

For this Reynolds, the topology of the flow changes with the aspect ratio of the device. As the aspect ratio of the channels increases, regions of high transverse velocities close to the vertical channels walls are formed (see green zones inside each of the channels). These regions enlarge and intensify with the aspect ratio, leading to higher relative velocities close to the walls and, consequently, to higher heat transfer values. This explains the trends observed in the right Figure 4 for moderate Reynolds.

**Figure 7:**
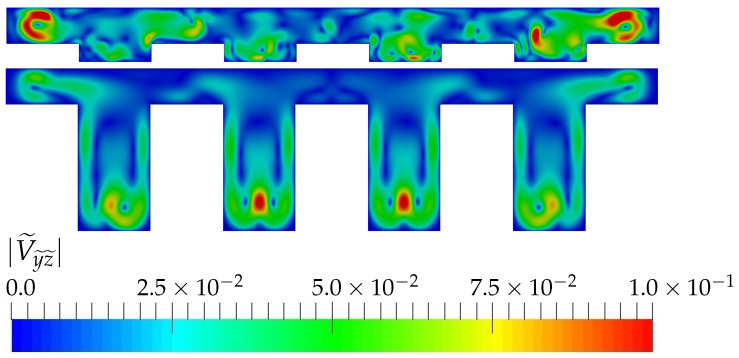
Transverse velocity at plane $S_{1}$ for Re=700 and TR=0.5 and different AR.

However, for high Reynolds, the topology of the flow changes. For small aspect ratios, the flow destabilizes to a unsteady solution, while for high aspect ratios the flow is still steady. Figure 7 shows the transverse velocity at plane $S_{1}$ for AR=0.25 and AR=1.75 for Re=700. The top figure, corresponding to AR=0.25, shows a snapshot of the transverse flow, where the symmetry of the flow in the span-wise direction is clearly broken, while for the bottom figure, corresponding to AR=1.75, the flow remains steady, symmetric and the transverse regions observed for lower Reynolds are still present. Therefore, the onset of destabilization is different and depends on the aspect ratio of the device. For the flow regimes investigated in this paper, this unstable regime observed for the smaller AR surprisingly degrades the heat transfer at the walls when compared with the higher aspect ratio devices. Therefore, the presence of the lateral-wall high transverse velocity regions are, again, responsible for the higher transfer rates.

## 4. Conclusions

The effect that different aspect-ratio channels have on the heat transfer of finned heat sinks with tip clearance is numerically studied. A 3D unsteady incompressible Navier-Stokes numerical model is solved in a simplified computational domain with four channels. In order to compare efficiencies between different devices with different cross-sections, the model is rendered dimensionless using the hydraulic diameter and the averaged flow velocity in the channel cross-section region. Flow boundary conditions were validated from previous experimental tests. Local and global Nusselt number, as well as the pressure drop, are computed once the numerical solution is obtained.

The results show that, for a given channel aspect ratio, the Nusselt number increases with the flow Reynolds number. For a given flow Reynolds number, the effect of different channel aspect ratios on the Nusselt shows two different behaviors: for moderate Reynolds, lower than 600 in the studied configurations, the heat transfer increased with the channel aspect ratio. As an example, for Re=400, the Nusselt multiplies by a factor of 1.14 between configurations AR 0.25 and 1.75 (Nusselt increases from 8.5 to 9.7, respectively). For moderate Reynolds, higher than 600, the maximum Nusselt is obtained for the squared-channel configuration. For Re=700, the Nusselt reduced by a factor of 0.89 and 0.98 for channel aspect ratios of 0.25 and 1.75, respectively, when compared with the squared-channel configuration. For all the studied cases, the (dimensionless) pressure drop decreased with the aspect ratio. In the context of micro-devices, where the Reynolds numbers are generally small, these results indicate that the use of channels with high aspect ratios is more beneficial, both in terms of thermal and dynamic efficiency. More studies should be done to extend the validity of this study to configurations for different combinations of tip clearance and aspect ratios, configurations which include more channels, and different Reynolds numbers.

## Figures and Tables

**Table 1:** Mesh sensitivity analysis for the case TR=W and Re=828.

Mesh	N° Cells	$\Delta \tilde{x}$	$\Delta \tilde{y}$	$\Delta \tilde{z}$	$\varepsilon_{1}$	$\varepsilon_{2}$
#1	$3.4\times 10^{6}$	0.10	0.04	0.04	6.2%	7.7%
#2	$7.7\times 10^{6}$	0.075	0.03	0.03	2.3%	1.4%
#3	$26.4\times 10^{6}$	0.05	0.02	0.02	Ref.	Ref.
